# A novel endoscopic recanalization strategy for refractory complete esophageal occlusion with pseudodiverticula after endoscopic submucosal dissection

**DOI:** 10.1055/a-2919-1298

**Published:** 2026-07-31

**Authors:** Zhen Tang, Jiamin Wang, Jiamin Qin, Li Wang, Liming Wen

**Affiliations:** 1 Department of GastroenterologyMianyang 404 HospitalMianyangSichuanChina; 2 Department of Science and TechnologyMianyang 404 HospitalMianyangSichuanChina

A 58-year-old male patient presented with progressively aggravated dysphagia and inability to take oral food after endoscopic submucosal dissection (ESD) for early upper esophageal carcinoma. Endoscopic balloon dilation was planned after thorough preoperative assessment. Intraoperative endoscopy revealed complete esophageal luminal occlusion 20 cm away from the incisors, along with postoperative cicatrization and two esophageal pseudodiverticula. Repeated cautious probing only detected a pinpoint orifice of about 0.2 cm, too narrow for a conventional dilation balloon to pass through. Under direct endoscopic vision, a zebra guidewire was inserted across the stenotic opening. The superficial scar tissue was pre-incised with an arch knife, and a 0.5-cm layered incision was then made with an IT knife along the physiological lumen to dissect the dense scar tissue and reconstruct the esophageal passage. Graded balloon dilation was subsequently conducted, achieving smooth endoscopic transit. The patient’s dysphagia was markedly alleviated postoperatively. No postoperative hemorrhage, perforation or other adverse complications were observed, and the patient was discharged with favorable recovery ( Video 1) .

**Video 1** A novel endoscopic recanalization strategy for refractory complete esophageal occlusion with pseudodiverticula after endoscopic submucosal dissection. Video URI: .


Conventional balloon dilation yields poor efficacy for complete esophageal occlusion following ESD, owing to rigid and non-compliant scar tissue. 1​ Concurrent pseudodiverticula further increases procedural risks, since blind operation may lead to mucosal laceration, perforation and mediastinal infection. We adopted a modified therapeutic regimen combining guidewire positioning, staged layered endoscopic incision and sequential balloon dilation. This approach successfully recanalized the fully occluded lumen using conventional endoscopic instruments without intraoperative complications. It remedies the limitations of single conventional balloon dilation and serves as a feasible and dependable alternative for refractory complete esophageal strictures after ESD.

Endoscopy_UCTN_Code_TTT_1AO_2AH
